# Inequality in the use of maternal and child health services in the Philippines: do pro-poor health policies result in more equitable use of services?

**DOI:** 10.1186/s12939-016-0473-y

**Published:** 2016-11-10

**Authors:** Karlo Paolo P. Paredes

**Affiliations:** Department of Healthcare Management and Policy, Graduate School of Public Health, Seoul National University, Building 221, room 411, 1 Gwanak-ro, Gwanak-gu, Seoul, Republic of Korea

**Keywords:** Inequality, Maternal and child health, Concentration index, Philippines

## Abstract

**Background:**

The Philippines failed to achieve its Millennium Development Goal (MDG) commitment to reduce maternal deaths by three quarters. This, together with the recently launched Sustainable Development Goals (SDGs), reinforces the need for the country to keep up in improving reach of maternal and child health (MCH) services. Inequitable use of health services is a risk factor for the differences in health outcomes across socio-economic groups. This study aims to explore the extent of inequities in the use of MCH services in the Philippines after pro-poor national health policy reforms.

**Methods:**

This paper uses data from the 2008 and 2013 Demographic and Health Survey (DHS) in the Philippines. Socio-economic inequality in MCH services use was measured using the concentration index. The concentration index was also decomposed in order to examine the contribution of different factors to the inequalities in the use of MCH services.

**Results:**

In absolute figures, women who delivered in facilities increased from 2008 to 2013. Little change was noted for women who received complete antenatal care and caesarean births. Facility deliveries remain pro-rich although a pro-poor shift was noted. Women who received complete antenatal care services also remain concentrated to the rich. Further, there is a highly pro-rich inequality in caesarean deliveries which did not change much from 2008 to 2013. Household income remains as the most important contributor to the resulting inequalities in health services use, followed by maternal education. For complete antenatal care use and deliveries in government facilities, regional differences also showed to have important contribution.

**Conclusion:**

The findings suggest inequality in the use of MCH services had limited pro-poor improvements. Household income remains to be the major driver of inequities in MCH services use in the Philippines. This is despite the recent national government-led subsidy for the health insurance of the poor. The highly pro-rich caesarean deliveries may also warrant the need for future studies to determine the prevalence of medically unindicated caesarean births among high-income women.

**Trial registration:**

Not applicable.

## Background

The advent of global development agendas have clearly pushed countries beyond limits to commit and invest in specific goals. One of these is the goal to reduce maternal deaths by three quarters between 1990 and 2015 (Millennium Development Goals). While the overall improvements in maternal health globally had been remarkable, the poor and the vulnerable were unfortunately left behind [1]. The Millennium Development Goals (MDGs) ended in 2015, but disparities in access to (and use of) maternal health services may still be observed in many countries. Post-MDGs, further reduction of maternal deaths remain in the recently launched Sustainable Development Goals (SDGs). Reducing the global mortality ratio to 70 deaths per 100,000 live births requires countries like the Philippines to scale-up efforts to effectively improve maternal and child health conditions. Evidence suggest improving Maternal and Child Health (MCH) services has an effect not only on the reduction of maternal deaths but extends to also impact reduction in neonatal and post neonatal mortality rates [2].

In the Association of Southeast Asian Nation (ASEAN) Region, five countries – Cambodia, Indonesia, Lao PDR, Myanmar and the Philippines, remain to have high maternal mortality ratio [3]. Among these five countries, the Philippines had the lowest annual change in reduction of deaths at 1.1 % from year 1990–2015 [4]. This low annual change in maternal mortality eventually led to the country not being able to achieve its MDG commitment to reduce maternal deaths.

In 2011, a Universal Health Care (UHC) strategy was launched in the Philippines [5]. In the strategy, attention was given to improving the overall health system and in protecting the poor from financial risks. A national government-led subsidy for the health insurance of the poor was not only seen as a means to increase healthcare utilization but also to ensure placement of sustainable healthcare financing [6, 7]. The maternal and child health strategy in the Philippines was also aligned with the launched UHC program – bringing the need to reduce unmet needs in family planning, facility-based deliveries and others in the national priority.

Many other reforms followed in the Philippines that is also expected to benefit the MCH situation. This includes the historical passing of a reproductive health law which aims to ensure government support to maternal and child health programs, together with the provision of modern contraceptives in government facilities [8]. Furthermore, the Conditional Cash Transfer (CCT) program in the country also includes conditions specific for maternal health. All women beneficiary of the cash transfer program is required to complete at least 4 antenatal care visits and deliver in a facility should they become pregnant [9]. While progress has been encouraging in the past decade, many other strategies are still needed to ensure protection of every pregnancy and children born in the country [10].

Theoretically, this study draws significant insights from Nyman’s work, emphasizing the access value of health insurance [11–14] and from Tudor Hart [15] on Inverse Care Law. Hart showed that the poor use lesser health services compared to its higher income counterpart, despite the poor having more needs for specific health services. Alternately, health insurance subsidies can increase use of health services among the poor (access value), thereby supporting the potential of observing a pro-poor shift in inequality. An increase in the use of health services, especially among individuals with low access to health services (e.g. the Poor) may not be necessarily inefficient (e.g. low-income women who will have better access to health facilities because of the health insurance). For MCH, deaths generally occur when women fail to receive the health services they need during and after the course of their pregnancy. Unless the inverse care law is resolved, women and children in low-income households will remain to be at high risk of dying from maternal complications. Pro-poor policies are obviously needed to help increase access of the poor to important maternal and child health services and reduce probability of deaths due to maternal complications. Further, other studies such as Andersen’ behavioral model for health service use also recognize health insurance as an important “enabler” for use [16, 17]. Health insurance, while definitely not the end solution to the problem of limited use of important health services among the poor, may provide a good ground for improving access to the needed MCH services [18].

Measuring the concentration index of maternal and child health care use in the Philippines before (2008) and immediately after (2013) extensive health insurance reform for the poor can provide good lessons in health policy. It has the potential to show whether a pro-poor policy reform has immediate short-term effect (pro-poor) on health care services use. Using data from two consecutive Demographic and Health Surveys (DHS) in the Philippines, this study focuses on key Maternal and Child health services use indicators.

## Methods

### Study setting/data used

This study uses data from the 2008 and 2013 Demographic and Health Survey in the Philippines [19, 20]. The DHS in the Philippines uses three questionnaires – Household, Individual women’s and Women’s safety module questionnaires. Response rate is high at 99 and 98.4 % in 2008 and 2013 respectively. Measured 5 years apart, the author predicts the 2013 DHS was able to capture the potential short-term effect of the pro-poor health policy reforms in 2011 on health care use. Maternal and child health services in the country are provided both by private and public entities, with the latter being mostly used by households in the lowest income quintile. History of MCH services use among women who gave birth one year preceding the DHS survey year was used.

Maternal and child healthcare use indicators utilized in this study are (a) complete Antenatal Care (ANC), (b) Facility-Based Deliveries (FBD) and (c) Caesarean-section deliveries (CS). Women with complete antenatal care is defined as mothers who in their last pregnancy, received all key components of antenatal care. Key components of ANC used in this study are the following: (1) Informed of signs of pregnancy complications, (2) weight measured, (3) height measured, (4) blood pressure measured, (5) urine sample taken and (6) blood sample taken. On the other hand, facility-based deliveries are births conducted in government or private birthing clinics or hospitals. Both the UHC and MCH policy in the Philippines promote use of facilities for deliveries to better manage intrapartum risks and eventually reduce deaths [5, 21]. Without adjusting for need (or medical indication), income-related inequality in caesarean deliveries was also measured to see if the recent policy reform insuring the poor changed rate of CS deliveries among households in the lower income quintile.

Other variables used are individual level demographic characteristics such as age, maternal education, women’s union status, wealth index, place of residence (urban/rural), and health insurance status. Household level variables used include the age and sex of household head and the number of household members. Geographic regions where the samples were collected was also used in the analysis.

Important in estimating inequalities, the author used the wealth index as computed by the DHS program per specific survey year. The wealth index is a factor score generated through principal component analysis, using specific household responses concerning household ownership of a number of consumer items [22, 23]. More information about the DHS wealth index construction for the Philippines in year 2008 and 2013 can be accessed through the DHS program site [24].

### Measurement and decomposition of inequality

Measuring inequality in health services use, methods advocated by Wagstaff, van Doorslaer and O’donnel was used [25–27]. Conceptually, these methods were developed in the context of the egalitarian principle of “equal treatment for equal medical needs” – exploring whether health care services are equally given to those who need it, regardless of differences in age, income and many others. However in the absence of Universal Health Coverage, as in the case many developing countries including the Philippines, adjusting for “need” is challenging [27, 28]. Without UHC, the egalitarian principle of equal treatment for equal need may not be satisfied.

Because of data and contextual limitations in the use of horizontal inequality index (concentration index adjusting to need) [28], Concentration Curve and Concentration Index (C) was used instead [25]. C is defined as twice the area between the concentration curve and the link of equality. In case where there is no socio-economic inequality, the concentration index is zero. However when the concentration curve lies above the equity line, it reflects a negative value, indicating a pro-poor inequality in health service use (in the context of this paper, indicates that more poor women delivered in a facility, received complete ANC services, etc.). Alternately, pro-rich use of services reflects a positive value [25]. The dependent variable *y* in this paper reflects health service use indicator as described. Using household survey microdata, the concentration index can be computed using the formula:

1 $$ C=\frac{2}{\mu }\ cov\left(y,r\right) $$

where μ is the mean of the health variable, *cov* as the covariance of *y* (health service use indicator) and *r* as the fractional rank (income rank variable) [25]. Further exploring determinants of inequalities, decomposing the C follows almost naturally. Decomposing the C as previously demonstrated by Wagstaff, van Doorslaer and Watanabe was used in this study [25, 29]. Exploring contributions of individual factors to the concentration index, the product of the sensitivity of health with respect to the individual factors and the degree of income-related inequality in that factor was determined. The linear additive regression of health (*y*) is expressed in Eq. 2.

2 $$ y=\propto +{\displaystyle \sum_k}{\beta}_k{x}_k+\varepsilon $$

3 $$ C = {\displaystyle \sum_k}\left({\beta}_k{\overline{x}}_k\ /\ \mu \right){C}_k+G{C}_{\varepsilon }\ /\ \mu $$

Decomposing the C for *y* factors, Eq. 3 follows. In the equation, ${\overline{x}}_k$ is the mean of *x*_*k*_, *C*_*k*_ is the concentration index for *x*_*k*_, and *GC* is the generalized concentration index for the error term (*ε*). In Eq. 3, the C is equal to the sum of the concentration indices of the *k* regressors. The residual component reflects the income-related inequality in health services use that is not explained by systematic variations in the regressors by income [25]. Despite the binary nature of the dependent variables, a linear probability model is used. Available literatures suggest decomposition of C shows no significant differences whenever linear and non-linear models are used [30, 31].

Further, the concentration curve of each dependent variables used is also shown. In figures, the concentration curve displays the share of health accounted for by cumulative proportions of individuals in the population ranked from poorest to richest [25]. The concentration curve was conveniently generated using MS Excel and the C and its decomposition computed using STATA 12 statistical software.

## Results

In absolute figures, women who delivered in health facilities increased from 2008 to 2013. Little change was noted for women who received complete antenatal care and caesarean births. As shown in Table 1, facility use for deliveries increased across all income groups. For those who are insured, use of facilities increased but so as for the uninsured where in absolute difference – the rate of increase is even higher. As for the rate of health services use according to the place of residence, use of maternal and child health services increased both in urban and rural communities with the former reaching at least 72 % of FBD in 2013.

**Table 1:** MCH services use by various maternal characteristics among births <1 year preceding the DHS, 2008 & 2013

Characteristic	DHS 2008 (%)			DHS 2013 (%)		
	ANC (CI)	FBD (CI)	CS (CI)	ANC (CI)	FBD (CI)	CS (CI)
Income status						
Poorest	6.76 (4.93–8.59)	12.83 (10.39–15.26)	0.55 (0.01–1.09)	11.32 (9.12–13.53)	35.35 (32.02–38.67)	1.26 (0.48–2.04)
Poorer	10.9 (8.33–13.46)	29.53 (25.77–33.27)	4.06 (2.43–5.69)	21.65 (18.19–25.11)	54.86 (50.68–59.05)	6.1 (4.08–8.12)
Middle	20.11 (16.01–24.21)	49.46 (44.34–54.47)	8.54 (5.66–11.42)	32.03 (27.63–36.42)	72.35 (68.14–76.57)	7.58 (5.05–10.11)
Richer	30.21 (25.32–35.09)	70.67 (65.83–75.52)	15.22 (11.37–19.08)	40.23 (35.03–45.43)	85.13 (81.36–88.9)	14.84 (11.03–18.64)
Richest	43.69 (36.9–50.48)	90.29 (86.24–94.35)	23.41 (17.6–29.23)	58.56 (52.06–65.06)	95.95 (93.34–98.55)	30.45 (24.36–36.55)
Age						
15–19	22.86 (15.87–29.84)	40.71 (32.54–48.89)	6.43 (2.35–10.51)	32.08 (24.79–39.36)	68.55 (61.31–75.8)	5.06 (01.63–08.49)
20–24	18.68 (15.36–22)	45.28 (41.03–49.53)	5.12 (3.24–7.01)	24.4 (21.02–27.78)	60.83 (57–64.67)	6.31 (4.39–8.23)
25–29	16.35 (13.47–19.23)	39.47 (35.66–43.27)	6.18 (4.3–8.06)	25.4 (21.98–28.81)	58.15 (54.28–62.02)	6.16 (4.25–8.06)
30–34	16.99 (13.59–20.38)	38.85 (34.44–43.26)	10.32 (07.55–13.09)	31.4 (27.26–35.55)	66.94 (62.74–71.14)	14.19 (11.04–17.35)
35–39	14.09 (10.08–18.1)	30.58 (25.28–35.89)	8.28 (05.1–11.45)	22.93 (18.27–27.59)	51.59 (46.05–57.13)	10.54 (07.13–13.95)
40–44	13.49 (7.5–19.48)	35.71 (27.31–44.12)	5.56 (1.54–9.57)	21.05 (13.53–28.57)	44.74 (35.56–53.91)	6.14 (1.71–10.57)
45–49	33.33 (8.63–58.04)	33.33 (08.63–58.04)	20 (−1.0–40.96)	26.32 (05.96–46.67)	52.63 (29.55–75.71)	0.00
PhiHealth						
Insured	24.3 (20.99–27.61)	58.36 (54.55–62.17)	13.15 (10.52–15.77)	28.97 (26.42–31.51)	64.08 (61.38–66.77)	10.19 (8.48–11.9)
Uninsured	14.14 (12.41–15.87)	31.54 (29.24–33.85)	4.69 (3.64–5.75)	23.37 (20.88–25.85)	55.15 (52.23–58.07)	6.25 (04.82–07.68)
Community type						
Urban	25.4 (22.50–28.31)	57.04 (53.74–60.34)	11.46 (9.32–13.6)	35.91 (32.80–39.01)	71.82 (68.91–74.73)	11.05 (8.99–13.1)
Rural	11.76 (10.04–13.49)	28 (25.59–30.4)	4.41 (3.31–5.51)	20.07 (17.99–22.16)	52.04 (49.44–54.64)	6.57 (5.28–7.86)
Union status						
in-Union	16.26 (14.69–17.84)	38.6 (36.52–40.68)	6.97 (5.88–8.06)	25.63 (23.8–27.46)	73.97 (66.83–81.12)	8.08 (7.02–9.33)
not in-union	35 (25.6–44.40)	56 (46.22–65.78)	11 (4.83–17.17)	36.3 (28.47–44.13)	58.87 (56.81–60.93)	10.27 (5.33–15.22)
Maternal education						
No Education	0.00	4.55 (−1.68–10.77)	0.00	5.26 (−1.94–12.46)	5.26 (−1.94–12.46)	0.00
Primary	6.7 (04.69–08.71)	15.08 (12.20–17.95)	1.35 (0.42–2.28)	11.3 (8.62–13.97)	36.85 (32.78–40.93)	2.25 (0.99–3.51)
Secondary	17.33 (15.05–19.60)	40.21 (37.26–43.16)	6.64 (5.14–8.15)	26.14 (23.64–28.64)	59.95 (57.16–62.74)	6.42 (5.01–7.83)
Higher	30.43 (26.42–34.45)	69.37 (65.34–73.39)	15.74 (12.55–18.93)	42.09 (38.05–46.13)	84.7 (81.75–87.64)	18.39 (15.21–21.57)
Sex of HH Head						
Male	16.17 (14.55–17.79)	37.88 (35.75–40.02)	6.85 (5.73–7.96)	24.61 (22.76–26.45)	57.8 (55.68–59.91)	7.81 (6.66–8.97)
Female	25.45 (19.73–31.17)	52.68 (46.12–59.24)	9.91 (5.97–13.85)	40.91 (34.70–47.12)	77.27 (71.98–82.57)	12.61 (8.38–16.83)
Age of HH Head						
< 50	15.31 (13.58–17.04)	34.72 (32.43–37.01)	6.26 (5.09–7.43)	22.98 (20.97–24.99)	55.11 (52.73–57.48)	6.93 (5.71–8.15)
50 and above	22.55 (19.05–26.04)	53.45 (49.28–57.63)	9.85 (7.35–12.35)	34.81 (31.16–38.46)	71.91 (68.46–75.35)	11.83 (9.34–14.31)

*ANC* Complete Antenatal Care

*FBD* Facility-based delivery

*CS* Caesarean Delivery

*CI* Confidence Interval

On the other hand in terms of maternal characteristics, the rate of maternal and child health care use increase with educational achievement. Women who are more educated tend to use more services compared to women who received little or no education. Union status reflect whether women are living with their partner or not. In the table, women who are not in union tend to receive better ANC and FBD compared to those who are in-union with their husbands. However, it should be noted that (1) majority of women in the Philippines are in-union (married and consensual union) hence the difference in the denominator maybe considered and that (2) the proportion are not causality indicators [19, 20]. While this relationship warrants a separate analysis, literature suggest that marriages and consensual unions are still protective of pregnancy [32, 33]. The case may also be the same with household characteristics. Majority of households in the country reports a head who is male (>80 % in 2008 and 2013 DHS).

### Concentration index

The concentration index of the selected indicators were computed using births accounted in less than a year preceding the DHS (2008 and 2013). Children born 1 year preceding the 2013 DHS reflects births that occurred after the health insurance subsidy for the poor was implemented. While changes in inequality may not be reflective of the overall impact of the insurance subsidy, a pro-poor shift may be looked at as an encouraging indicator. In the Philippines, the pro-poor UHC policy was initiated to help increase use of services among the poor [7].

**Fig. 1:**
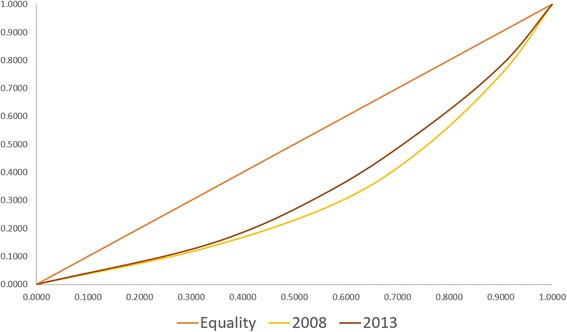
Concentration Curve of the utilization of complete ANC services for births <1 year preceding the DHS

**Fig. 2:**
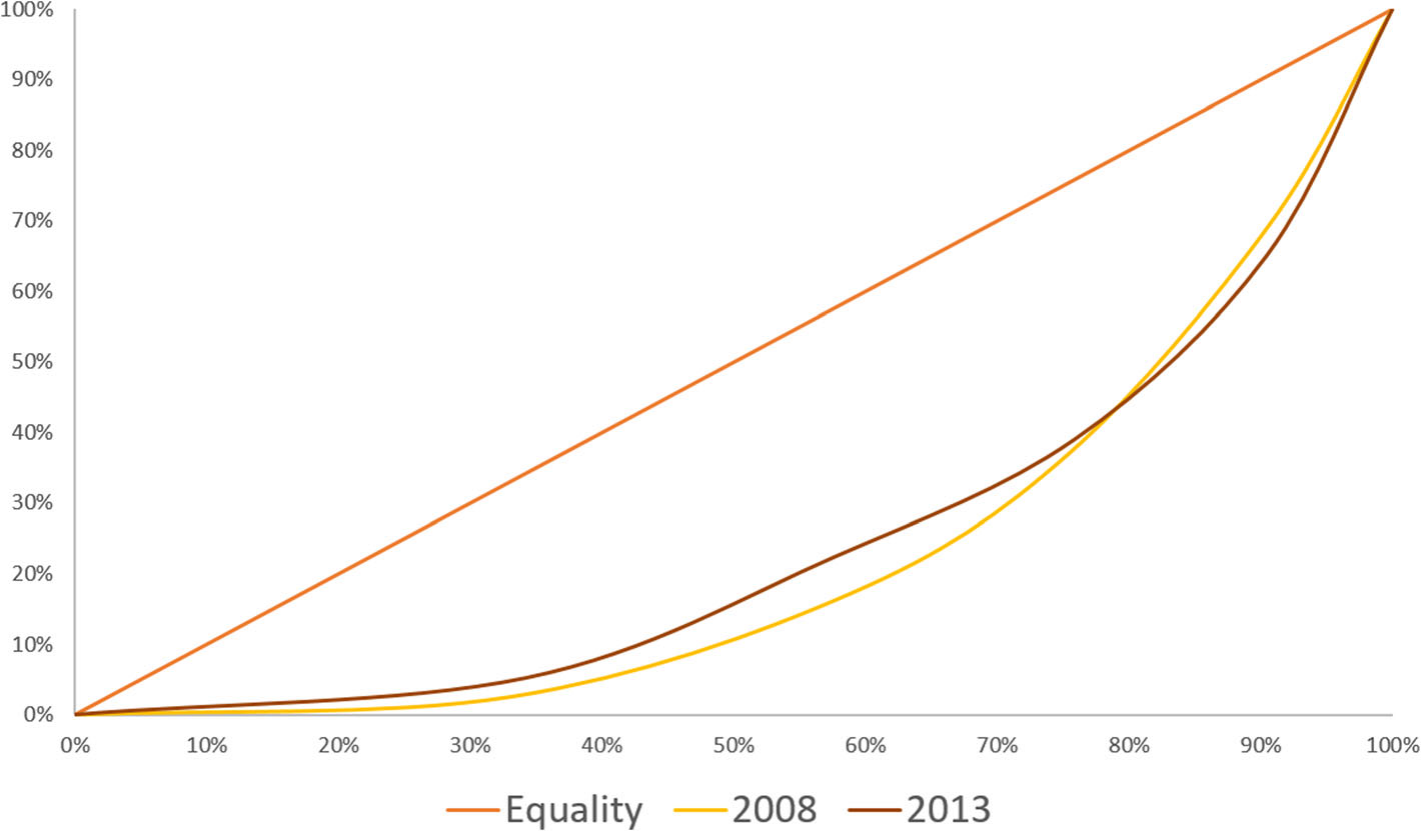
Concentration Curve of the Caesarean Deliveries for births <1 year preceding the DHS

The results show that for ANC, FBD and CS, use of services tend to favor the rich. However, improvements in inequality can be noted, especially for facility-based deliveries. The computed concentration index for ANC services and CS births in 2011 and 2013 did change a little but remains highly pro-rich (Tables 2 and 4; Figs. 1 and 2). For complete ANC, a slight improvement can be noted while for CS, a slight shift can only be seen in the concentration curve but the index generally remains the same (C > 0.5).

**Table 2:** Concentration index and decomposition of complete ANC use from births <1 year preceding the DHS, 2008 and 2013

	Complete antenatal care use			
	2008		2013	
	Contribution	%	Contribution	%
Concentration Index	0.3814		0.3206	
PhilHealth Membership	0.0150	3.93 %	0.0025	0.78 %
Age	−0.0025	−0.66 %	−0.0014	−0.44 %
Wealth - q2	0.0004	0.12 %	−0.0047	−1.46 %
Wealth - q3	0.0178	4.66 %	0.0207	6.44 %
Wealth - q4	0.0676	17.73 %	0.0555	17.32 %
Wealth - q5	0.0974	25.53 %	0.0944	29.44 %
Education	0.0798	20.94 %	0.0625	19.51 %
Union Status	0.0054	1.41 %	−0.0008	−0.25 %
Place of Residence	0.0116	3.03 %	−0.0028	−0.88 %
Age of HH Head	0.0200	5.25 %	0.0216	6.72 %
Sex of HH Head	0.0000	0.01 %	0.0060	1.87 %
Number of HH Members	−0.0029	−0.76 %	−0.0001	−0.03 %
Region fixed effects	0.0624	16.37 %	0.0618	19.26 %
Residual	0.0093	2.44 %	0.0055	1.71 %

**Fig. 3:**
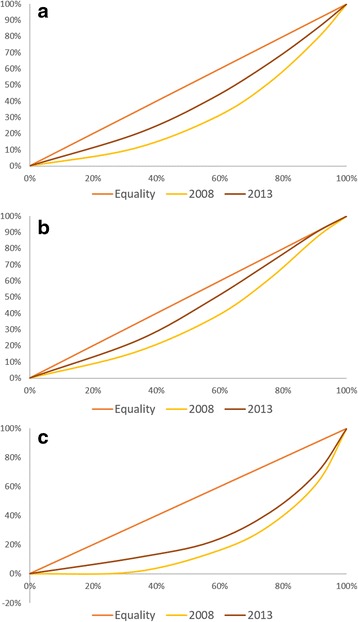
**a** Concentration Curve of the utilization of appropriate facility for delivery for births <1 year preceding the DHS (Government and Private). **b** Concentration Curve of the utilization of appropriate facility for delivery for births <1 year preceding the DHS (Government). **c** Concentration Curve of the utilization of appropriate facility for delivery for births <1 year preceding the DHS (Private)

The concentration index for facility-based deliveries was computed with disaggregation by ownership type. This is to allow for comparison and see if improvements in inequalities can be noted in both government and private health facility use. Comparing the computed 2008 and 2013 concentration index, deliveries remains generally pro-rich in both government and private facility but have become less in 2013 compared to 2008 (pro-poor shift). Government facilities have become more pro-poor (CI = 0.1217 in 2013) compared to private facilities (C = 0.4757 in 2013). Table 2 and Fig. 3 shows the concentration index and concentration curves for facility deliveries in years 2008 and 2013.

### Decomposition of the concentration index

In the same table where the concentration indexes are presented (Tables 2, 3 and 4), decomposition using selected variables are also shown. The rate of contribution of each factor is accounted to each concentration index as computed. Factors used include income, insurance status (PhilHealth membership), women and household characteristics, and region fixed effect. The region fixed effects represents the inequality attributable to the region where the sample was collected. Population, geographic and socio-political characteristics of regions in the Philippines are heterogeneous. Distribution of health facilities are also uneven, being it concentrated mostly in highly populated localities. The residual reflect the contribution of factors other than those included in the estimators used.

**Table 3:** Concentration index and decomposition of the use of appropriate facilities from births <1 year preceding the DHS, 2008 and 2013

	Deliveries in Government and Private				Deliveries in Government Facilities				Deliveries in Private Facilities			
	2008		2013		2008		2013		2008		2013	
	Contribution	%	Contribution	%	Contribution	%	Contribution	%	Contribution	%	Contribution	%
Concentration Index	0.3777		0.2128		0.2666		0.1217		0.5877		0.4757	
PhilHealth Membership	0.0205	5.44 %	0.0018	0.86 %	0.0012	0.45 %	−0.0013	−1.08 %	0.0571	9.71 %	0.0109	2.29 %
Age	0.0012	0.32 %	0.0003	0.13 %	0.0026	0.98 %	0.0003	0.21 %	−0.0014	−0.24 %	0.0003	0.06 %
Wealth - q2	−0.0052	−1.37 %	−0.0043	−2.03 %	−0.0032	−1.20 %	−0.0055	−4.51 %	−0.0089	−1.51 %	−0.0010	−0.20 %
Wealth - q3	0.0295	7.81 %	0.0207	9.71 %	0.0267	10.02 %	0.0180	14.77 %	0.0347	5.90 %	0.0284	5.97 %
Wealth - q4	0.0933	24.72 %	0.0524	24.64 %	0.0711	26.66 %	0.0311	25.52 %	0.1355	23.05 %	0.1141	23.99 %
Wealth - q5	0.1122	29.70 %	0.0565	26.54 %	0.0285	10.70 %	0.0012	0.97 %	0.2703	45.99 %	0.2161	45.43 %
Education	0.0658	17.42 %	0.0434	20.40 %	0.0503	18.88 %	0.0454	37.30 %	0.0951	16.18 %	0.0376	7.91 %
Union Status	0.0010	0.25 %	0.0000	−0.01 %	−0.0001	−0.05 %	0.0015	1.20 %	0.0030	0.51 %	−0.0043	−0.90 %
Place of Residence	0.0254	6.72 %	0.0063	2.97 %	0.0425	15.95 %	−0.0066	−5.38 %	−0.0070	−1.20 %	0.0435	9.14 %
Age of HH Head	0.0147	3.89 %	0.0084	3.95 %	0.0181	6.78 %	0.0084	6.92 %	0.0083	1.41 %	0.0083	1.75 %
Sex of HH Head	−0.0018	−0.49 %	0.0024	1.14 %	0.0020	0.73 %	0.0023	1.85 %	−0.0090	−1.53 %	0.0029	0.61 %
Number of HH Members	−0.0016	−0.43 %	−0.0001	−0.03 %	−0.0008	−0.31 %	0.0000	−0.02 %	−0.0031	−0.53 %	−0.0002	−0.03 %
Region fixed effects	0.0124	3.29 %	0.0183	8.58 %	0.0122	4.57 %	0.0237	19.48 %	0.0129	2.19 %	0.0025	0.53 %
Residual	0.0103	2.73 %	0.0067	3.16 %	0.0155	5.83 %	0.0034	2.76 %	0.0004	0.06 %	0.0164	3.46 %

**Table 4:** Concentration Index and decomposition of CS Deliveries from births <1 year preceding the DHS, 2008 and 2013

	CS Deliveries in Government and Private			
	2008		2013	
	Contribution	%	Contribution	%
Concentration Index	0.5514		0.5162	
PhilHealth Membership	0.0417	7.56 %	0.0014	0.28 %
Age	−0.0106	−1.93 %	−0.0003	−0.06 %
Wealth - q2	−0.0045	−0.82 %	−0.0110	−2.12 %
Wealth - q3	0.0297	5.38 %	0.0251	4.86 %
Wealth - q4	0.1278	23.18 %	0.1160	22.48 %
Wealth - q5	0.1877	34.04 %	0.2620	50.76 %
Education	0.0807	14.64 %	0.0751	14.56 %
Union Status	0.0024	0.44 %	0.0037	0.72 %
Place of Residence	0.0103	1.87 %	−0.0236	−4.58 %
Age of HH Head	0.0254	4.60 %	0.0166	3.21 %
Sex of HH Head	−0.0075	−1.36 %	−0.0014	−0.27 %
Number of HH Members	−0.0056	−1.01 %	−0.0002	−0.04 %
Region fixed effects	0.0665	12.05 %	0.0509	9.86 %
Residual	0.0074	1.35 %	0.0017	0.34 %

The results show that the concentration index for all indicators are influenced predominantly by household wealth. Especially for ANC and CS, the contribution of household wealth even increased in 2013 when compared to 2008 (ANC = 48.03 % in 2008 and 51.74 % in 2013; CS = 56.48 % in 2008 and 80.29 % in 2013). For FBD, the contribution of household wealth decreased at different rates when facility ownership type is considered. The effects of household wealth also increased in 2013 for private facilities (73.43 % in 2008 and 75.18 % in 2013) but decreased in government facilities (46.18 % in 2008 and 36.75 % in 2013) eventually leading to decreased contribution of income overall (60.65 % in 2008 and 58.85 % in 2013). Nonetheless despite the decrease, the contribution of household wealth to inequality in facility-based deliveries is still large (>50 %).

**Fig. 4:**
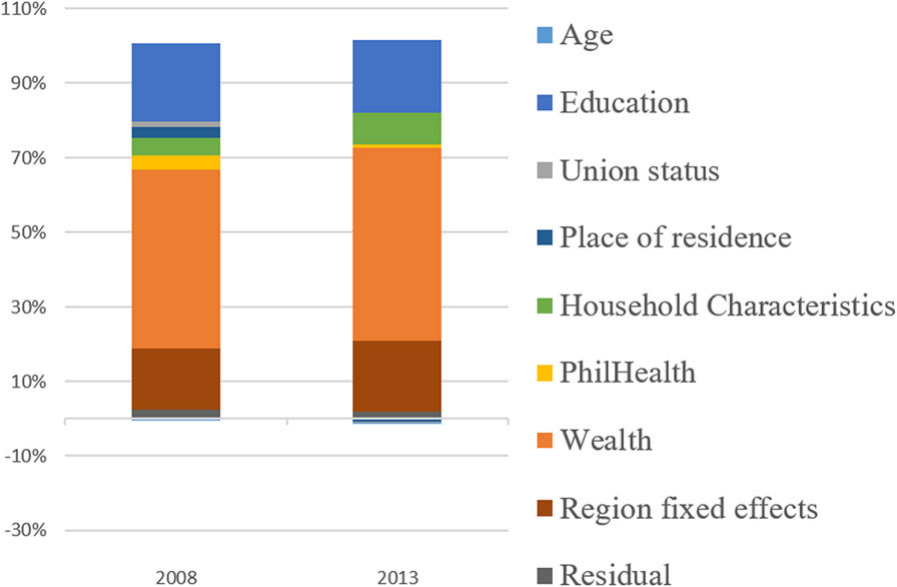
Decomposition of Inequality in utilization of complete ANC services for births <1 year preceding the interview

**Fig. 5:**
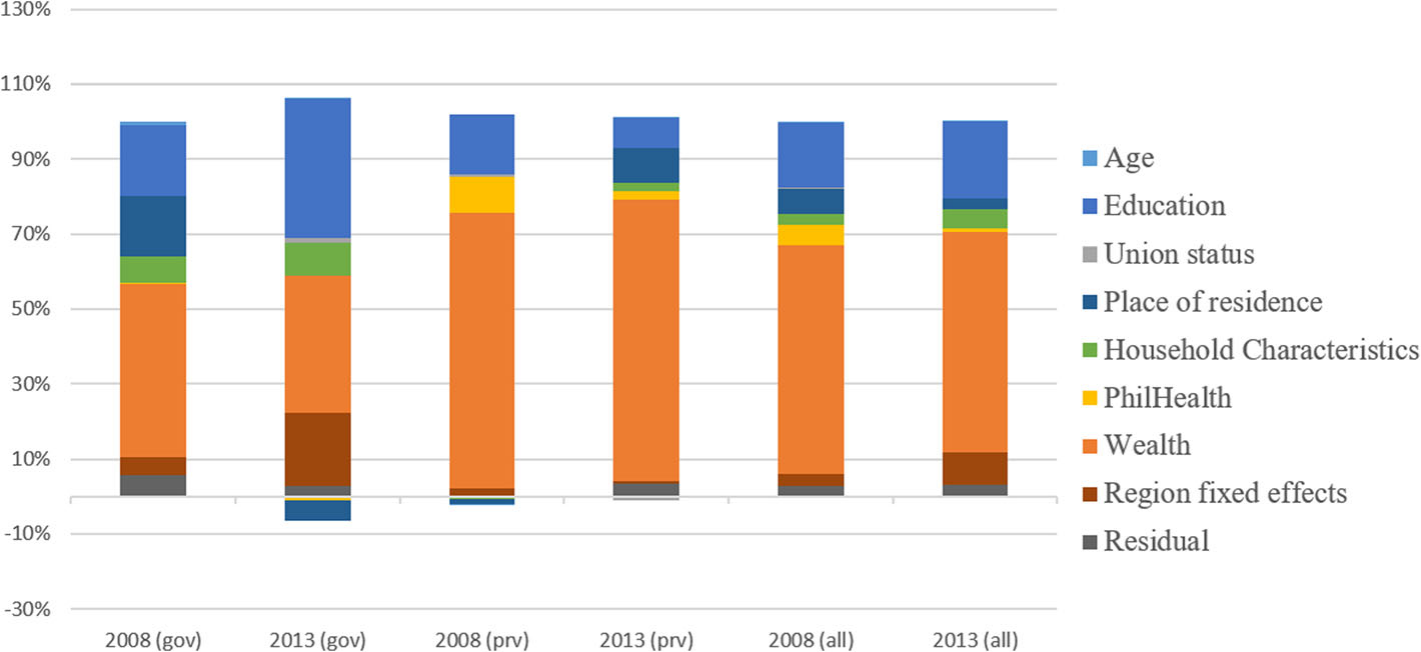
Decomposition of Inequality in utilization of appropriate facilities for delivery for births <1 year preceding the interview

**Fig. 6:**
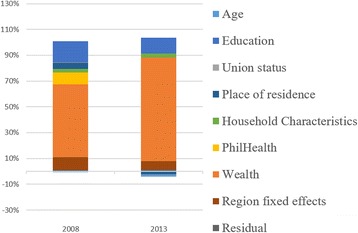
Decomposition of Inequality in utilization of complete ANC services for births <1 year preceding the DHS

Following household wealth, women’s education contribute to inequities the most across all selected indicators (In 2013: 19.39 % for ANC; 20.40 % for FBD; 12.34 % for CS). For complete antenatal care services use, the contribution of region fixed effect is highest at 16.37 % in 2008 and 19.26 % in 2013. Moreover for FBD, Regional differences also notably increased for deliveries in government facilities from 4.57 % in 2008 to 36.75 % in 2013 leading to the slight increase overall in region effects (3.29 % in 2008 to 8.48 % in 2013). The contribution of health insurance (PhilHealth) membership to inequalities was lower in 2013 compared to 2008 (Tables 2, 3 and 4). This is despite the expanded coverage of health insurance among the households in the lower income quintile. Details of the contribution of other factors in the decomposition analysis can be found in Tables 2, 3 and 4 and Figs. 4, 5 and 6.

## Discussion

Pro-poor health policy reforms in the Philippines have clearly expanded health insurance coverage but equal treatment for equal medical needs are yet to be achieved. Improving inequalities in the use of MCH services have the potential of not only improving the overall health of women and children but also in reducing probability of deaths from maternal causes associated with non-use of health services. Inequalities in health care use remain pro-rich, but the recent a pro-poor improvement, especially for facility-based deliveries is encouraging to further support future pro-poor health policy reforms.

Measuring income-related inequity in maternal and child health care use, this paper used the method as advocated by Wagstaff, van Doorslaer, O’donnel, et al. [25, 27, 28]. Concentration Index as a summary indicator can be used to show income-related inequalities in health or utilization of health care services. Extensively used to model inequalities in high-income countries, the concentration index may also be adjusted to differences in need. Horizontal Inequality Index (HI) is a concentration index that adjusts for the differences in need across income quintile group [31, 34]. Using HI, the following assumptions should be met: (1) universal coverage is already achieved (as what is usually the case in high-income countries) and (2) a proxy for health care need (e.g. self-rated health measure) is available. However in the context of low-income countries in Asia including the Philippines, these two assumptions are still not satisfied. Many if not all, are still developing and/or implementing strategies to achieve universal coverage. The use of self-rated health measures may be limited in the context of low-income countries since they are most probably unavailable or unreliable [27]. Baseline study in the Philippines and other low-income countries suggest a highly pro-rich inequality in the use of health services [35]. When we consider the inverse care law, a pro-poor use is equitable, even without adjusting for need [27, 35, 36]. When health facility visits are mostly influenced by income or capacity to pay, the principle of facility use based on needs or “equal treatment for equal need” may not be observed [27].

Using the most recent national survey yet in the Philippines, inequality in the use of MCH services remain pro-rich after extensive subsidy for the health insurance of the poor. The large and increasing contribution of household wealth (>50 %) shows that the use of health care services in the country is still driven largely by differences in income. For ANC and CS deliveries, little has changed from 2008 to 2013. Interestingly for complete ANC use, the figures may be used to suggest the need to re-evaluate strategies for ANC. The increasing frequency of ANC visits in the country should lead to more women receiving complete care (2013 DHS notes 84 % of women have four or more ANC visits in the Philippines). Otherwise, women who would have complete antenatal care will be left only to those who can afford it (e.g. Women who can bear the cost of blood examination, etc.). Household wealth, maternal education and regional disparities were the major drivers of inequities in antenatal care. These findings are consistent with available literatures in the country exploring determinants of antenatal care use [37–40].

On the other hand while still pro-rich, inequalities in facility-based deliveries improved the most (Fig. 3). Further disaggregation of use by ownership type shows a pro-poor shift for both government and private facilities. This change in inequality in the use of health facilities for delivery indicates increase in use among women in the lower income bracket. The decreasing contribution of income to the concentration index of facility-based deliveries in 2013 may also be found encouraging, especially since the subsidy for the health insurance of the poor has just been introduced. For deliveries in government facilities, the increasing contribution of regional differences may also indicate the need to review the distribution of functional health facilities across regions. In cases where birthing facilities are inaccessible to the poor (because of limited functional facilities), income-related inequalities in maternal and child health services use may vary [10]. Nonetheless despite the noted improvements, household wealth remains to be a strong contributor to the inequalities in FBD observed.

Caesarean deliveries remain highly pro-rich in the Philippines. Estimates did not change much from 2008 to 2013 despite PhilHealth coverage for CS deliveries and the noted increase in PhilHealth membership among the poorest households [41, 42]. The contribution of income to the resulting inequality in 2013 even increased, the highest compared to other indicators accounting to 80 %. Despite the increasing contribution of income to inequalities in CS deliveries, the rate of CS births did not change much from 2008 to 2013 [19, 20]. Since medically unindicated CS deliveries are now becoming a concern worldwide, the resulting inequality in this study may provide a ground for future studies exploring this practice among the higher income group [43, 44]. Further studies to establish whether CS deliveries are conducted in the Philippines without medical indication, and to what extent this is driving inequalities in caesarean deliveries is recommended.

Supporting the implementation of strategies to achieve UHC in the Philippines, this study shows the importance of monitoring equality in the use of MCH services. Pro-poor policies are still needed, especially since use of health services in the Philippines remain highly pro-rich. As recognized in the recent MDG evaluation in the Philippines, it is important to make sure that women, regardless of their income status, will be able to use their needed MCH services [10]. It may also be desirable if the country adjusts its reporting practices to include disaggregation by income groups – and compare utilization rates of health services between the rich and the poor. Monitoring of progress towards reducing pro-rich inequalities using viable data could reinforce the need to reduce income-related inequality. Increasing investments to protect the most disadvantaged households (e.g. insurance for the poor) and other demand side interventions to make sure the poor is able to use their needed health services should nonetheless be supported to promote more equitable use of health services [45].

Important limitations are also noted in this study. First is the need to establish if the recent reforms were indeed the reason for the increase in use of MCH services, especially among the poor. The methods used in this study does not identify whether the increase was specifically because of the recent reforms initiated (e.g. health insurance effects). Second is the limitation in the timing of the available national survey in the Philippines. The 2013 DHS was conducted only 2 years after the extensive reforms was made in the country. The estimates derived in this study may not show the complete picture of the full potential of the pro-poor policy reforms initiated in 2011. Lastly is the recognition of the need to adjust for “need” in the CS estimates. Unlike the complete ANC and facility-based deliveries, medical indications for CS deliveries may further expose inequalities in its use. Until such time need variables for CS deliveries becomes available, the results of this study is still useful. Continuous monitoring of inequality in MCH services use is important to support health system interventions that are pro-poor and are beneficial to promote a more equitable use of MCH services in the Philippines.

## Conclusion

Historically in the Philippines, health care use has been pro-rich. Previous estimates and this study showed that this phenomenon remains true using the 2013 data of the DHS. The recent reforms expanding health insurance for the poor may provide a good vehicle to improve the observed inequalities. However as noted in this study, its effect on reducing inequalities may not be observed fully in short-term. Further studies related to inequalities may be warranted to drive more pro-poor health policy reforms until the country achieve equal treatment for equal need. Programmatically, the large contribution of household wealth to the inequalities observed should be reduced. This can be done by continuing the institution of health policies that will facilitate use of health services according to need and not on individual’s or household’s capacity to pay for health services. The noted improvements in facility-based deliveries among the poor may also be complemented with strategies that will make sure regional disparities in access to health care facilities will be reduced. Further, the need to improve access of the poor to complete antenatal care use also shows the importance of the need to not only increase use of health services (number of antenatal care visit) but also to improve the quality of health services provided in health facilities (provision of complete antenatal care). The resulting inequalities further suggest the need to continue pro-poor health policy reforms as the country adapts to the targets set in the recently launched Sustainable Development Goals.

## Abbreviations

ANC: Antenatal care
C: Concentration index
CC: Concentration curve
CS: Caesarean-section deliveries
DHS: Demographic and health survey
FBD: Facility-based deliveries
HII: Horizontal inequality index
MCH: Maternal and child health
MDG: Millennium development goal
NBB: No balance billing
UHC: Universal health care
